# Some Types of Heart Disease

**Published:** 1882-01

**Authors:** W. D. Bizzell

**Affiliations:** Atlanta, Ga.; Professor of Chemistry in Southern Medical College


ATLANTA

### MEDICAL REGISTER.

VOL. I.] JANUARY, 1882. [No. 4

### Original.

### SOME TYPES OF HEART DISEASES.—A CLINICAL LECTURE, BEFORE THE CLASS, AT SOUTHERN MEDICAL COLLEGE.

BY W. D. BIZZELL, M.D., ATLANTA, GA.
Professor of Chemistry in Southern Medical College.

*Gentlemen*—We have been peculiarly fortunate recently in presenting, for your consideration, three cases of heart disease, which may be regarded as typical, and serve excellently well to illustrate the more important classes of disease you will meet with in practice, affecting this vital organ.

The first case that claims our attention is that of Mr. W. L., a gentleman 35 years of age, who comes to us from one of the upper counties in this State. There is no evidence of anæmia present, though the patient could hardly be classed as vigorous, being rather thin and spare, with only moderate chest development. His history, as detailed in your hearing, is that for the past three years he has suffered more or less from palpitation, at times quite violent, attended not unfrequently by a feeling of weight, oppression and painful constriction in the precordia. Between the paroxysms no pain or inconvenience was felt, and he continued to pursue his vocation as a farmer, but the symptoms continually grew worse. Under the advice of his local physician the use of tobacco was abandoned, his habits as to work were regulated to avoid excessive fatigue, and attention to diet and correction of digestive derangements, then existing. He says, further, that he has taken digitalis with good effect. On examination we find his pulse is normal in frequency, tone and rythm; on inspection we find the apex beat in its normal position, on a line with the nipple and one inch below. Careful auscultation, with only the under shirt intervening, show the heart sounds normal in all particulars. A further auscultation of the chest shows that there is no aneurism or constriction of the aorta. No tumor deep in the sub-clavicular or lower cervical region which could compress or disturb the important nerve trunks that underlie this area; so far as we can judge, by the most rigid examination, there are no marked physical changes. We say, here is a case of functional disturbance, or we might use the really unintelligible phrase, functional disease; meaning thereby that there are no coarse textural changes to account for the symptoms exhibited, not but there may be subtle changes of the delicate component cells which even the far reaching eye of the microscopist fails to detect. As the cardiac muscle shows apparently no continued or permanent weakening following these outbursts of violent or purturbed action, by common consent the nervous system is believed to be at fault, hence these symptoms are grouped together under the general head of cardiac neuroses.

Hence, before we can intelligently appreciate the nature of these symptoms, or afford rational treatment, it is absolutely necessary that we make a careful study of the nerve supply of this delicately poised, vital pump; also the relations which it sustains to the nerve supply of the important organs which lie adjacent to it. First in importance, then, is the pneumo-gastric. This wonderful tenth pair of the cranial nerves arise from the medulla-oblongata by a series of ten or fifteen filaments in a continuous series; the nucleus, from the deeper part of which these fibres take their origin, is an extended tract of gray matter known as the area cinerera, extending along the posterior surface of the medulla-oblongata, just outside of the lower extremity of the faciculus teres. These filaments unite in a single trunk, and in company with the spinal accessory and glosso-pharyngeal pass out through the cranium to jugular foramen; at this point there is a ganglion, the jugular ganglion, at or just beyond this point it is joined by motor branches from the spinal accessory and a small branch from the facial and below this point it receives branches from the hypoglossal and the anterior trunks of the first and second branches of the cervical nerves. In the upper cervical region it gives off branches which form the pharyngeal plexus, and by this plexus it receives its first link with the great sympathetic nerve. Giving off the superior laryngeal plexus, which is a sensory nerve, and the inferior laryngeal which is a motor nerve. Just below this point are given off the two principal cardiac nerves of the pneumogastric. Passing down into the chest the pulmonary plexus, formed by the inosculation of the branches of the nerves on either side, which by their subdivisions supply the mucous surfaces of the various divisions and subdivisions of the bronchi, giving off at the same time a plexus which surrounds the œsophagus; in this portion of its course it also sends by way of the sympathetic numerous branches to the heart, the centre of circulation. Reuniting below the pulmonary plexus, the pneumogastrics penetrate the diaphragm and spread out in two sets of gastric branches, which supply the mucous membrane and muscular coat of the stomach. Those from the left supply the anterior wall of the stomach as far as the pylorus, and along with the sympathetic send branches through the fissure of the liver, which are distributed throughout the substance of that organ. Branches from the right are distributed to the posterior wall of the stomach, and finally communicate with the solar plexus of the sympathetic.

This important pair of nerves, therefore, are distributed to the mucous surfaces and muscular structure of the passages for the introduction of air and food into the organism, the stomach, liver, and by inoscutation with the sympathetic to the heart, and the radiating sympathetic plexus of the abdominal organs.

The root of the pneumo-gastric at its point of origin is purely a nerve of sensation, but motor influence is communicated to it from the facial, the spinal accessory and cervical nerves. The physiological effect produced by section of the pneumo-gastrics in the middle of the neck is an immediate and progressive slowing of the respiratory movements and increase of the heart's action. The progressive slowing of the respiration continues till the act ceases altogether. Death ensues from lack of aereation of the blood, though the heart continues for a time to beat after the cessation of respiration.

Beside the nerve branches which we have mentioned as coming directly from, and indirectly through the sympathetic, from the pneumo-gastric to the heart, other branches pass directly from the stellar plexus and the great solar plexus to this organ, and even in the muscular structure of the heart itself near the auriculo-ventricular septum on either s de, are ganglia capable of producing cardiac contraction by proper stimulation, even after the heart has been removed from the body for hours. Specially is this true of the lower orders of animal life, the heart of the turtle, for instance, which, under the stimulus of electricity or injections into its cavity of warm blood, will not unfrequently respond by contraction twenty-four hours after its separation from the body.

The physiological action of the various nerves which we have mentioned are peculiar, diverse, and on the proper performance of their functions depend those rythmical movements of the vital organs essential to the continuance of life itself. The pneumo-gastrics not only supply the upper part of the larynx with sensitive fibres which guard the rima-glottidis against the introduction of foreign bodies, and the lungs with sensation fibres which convey to the respiratory centre in the medulla the impression that there is blood in the lungs demanding aeration, a sensation which even the will power is unable to restrain, thus insuring the continued performance of the vital act of respiration, but send directly and indirectly fibres to the heart which exert a powerful influence on the functional action of this organ. The fibres, which uniting form the right and left trunk of this nerve for distribution to the stomach, seem to be motor, and originate and control those rythmical, churning movements which are essential to digestion, not directly controlling the gastric secretion, but by continual stimulation of the gastric mucous membrane by successive contact with food, cause an abundance of the gastric juice to be poured forth.

From the fact that the fibres of the pneumo-gastric supplied to the heart are in such intimate and close relations with the sympathetic of the chest and abdomen, and the great difficulty of determining by physiological experiment the function of the sympathetic, make it a difficult matter to determine the precise influence which each nerve exerts on the muscular walls of the heart to produce its rythmical movements, or exactly which link in the chain of inervation is broken when irregularity of movement or inefficiency of action is manifested.

We know this much, however, that stimulation of the trunks of the pneumo-gastric by a strong current of electricity causes a slowing of the pulse-beat, and if the impression be sufficiently strong, pulsation may be arrested altogether and death result in diastole; further, if the pneumo-gastrics be divided in the middle of the neck, as we have previously stated, the heart's action is immediately increased, accelerated; from this experiment physiologists have denominated the fibres of the pneumo-gastric which are distributed to the heart as inhibitory, or controlling and regulating. And that in man, at least, they perpetually exert this inhibitory or repressing action, and when their influence is removed by division of their trunks, there is a rebound as it were, and another set of nerves, the accelerators, take control of the heart's movements, with the results already stated.

An acceleration centre is believed by some physiologists to be in the medulla oblongata, but its exact locality they have not yet been able to tell us. Irritation of the medulla-oblongata causes an accelerated pulse-beat and contraction of terminal arterioles, provided the heart be not cut off from the spinal cord and the rami-communicantes of the stellar plexus be intact.

The action of certain poisons throw light on the determination of this difficult question.

Muscarine, the active alkaloid principle obtained from the amanita muscaria, when administered either hypodermically or internally, causes a remarkable slowing, and in sufficient quantity, ultimate stoppage of the heart's action in diastole, and section of the pneumo-gastrics does not prevent this action. Atropia, on the other hand, causes the heart to beat more quickly and interferes with the effect produced by irritation of the vagus, and counteracts the toxic effect of muscarine. It is not sufficient to say that these effects are produced by irritation or paralysis of the inhibitory vagus endings, for nicotine, which acts like atropia (though its immediate effect after administration seems to be exactly the reverse), yet does not prevent the stoppage of the heart from muscarine. Nicotine seems to paralyze the inhibitory termini of the vagi, while muscarine and atropia act through their influence on the inhibitory centre of the medulla. The exact route by which the normal rythmic nervous impulses travel to the cardiac muscles is not as clear as we might desire; or how morbid impressions reach the heart and destroy its harmony, so that this muffled music of life becomes jangled and out of tune. But in addition to the experimental fact furnished by the physiologist. the clinical experience of daily practice teaches that occasionally this ebb and flow of the nervous influence is eliminated irregularly, and an irregular pulse beat and heart action is congenital and is not at all incompatable with good health or even comparative longevity.

Also, in that state called hysterical, where the inhibitory control apparently of the sympathetic nervous system is at fault, and impresses itself on the faculties of intellection as a weakening of the will power, the nervous impulses controlling the hearts action run riot and we may have palpitation, irregularity and pain referred to the cardiac region. Others peculiarly susceptible to the influence of nicotine, succumb quickly to its morbid influence and cannot use tobacco even in small quantities without suffering severely. Others only suffer after a long period of excessive use of the weed. Others by habitual and persistent gormandising, so overtax and weaken the digestive powers that the nervous system sympathizes with the patient and much abused stomach and functional disturbances of the heart declare themselves as a part of the penalty inflicted by outraged nature. Exhaustion of the nervous force, whether it be from engrossing brain work or nervous excitements of a lower order, as the wear and tear of worry and friction which some indulge in, or the grosser passion of venery; whatever it may be that produces these functional disturbances you will invariably find a state of impaired digestion and imperfect assimilation, either antedating or marking the outbreak of the apparently more serious symptoms for which you are consulted. The painful disorders grouped under the head of neuralgia, unless due to mechanical causes, rarely declare their presence, except there be a state of anæmia or malnutrition; but if the painful impression and morbid action of the nervous system by frequent repetition has become a vicious habit then, even after good nutrition has been established, the nerves will, if submitted to extra strain, again discharge their force in the old morbid channels and the old pain asserts itself. The same is true of epilepsy and insanity and other diseases of the nerve centres; and we cannot expect the heart neuroses to be an exception to the rule, and once the morbid action is fixed upon the person, a weakness in this direction and a liability to return must ever be remembered, and the patient cautioned against the cause which originally excited the disorder.

In the way of treatment we must be guided by the history and present appearance and state of the patient, guard him against the needless waste of his vital energies from whatever cause, see that his habits of eating, drinking and living are properly regulated. In the patient before you these indications have been very properly met by his local medical adviser; and as to medicine, I would suggest the tinct. of nux vom. and tinct. chlor. iron—m. v. of the former and m. x of the latter, three times a day in water.

To allay the intensity of the paroxysms during the attack, many remedies have been suggested. Some simple sedative as brom. potass. and valerian, ammo. tinct. of valerian. The fld. ext. of cactus grandiflorus and the cactus bonplandi have been highly commended, and the few times they have been used by me they seemed to do good, and to quiet the heart's action.

The question as to the danger of these so called functional diseases will be often demanded of you by the patient and his friends. After careful examination, if you detect no sign of organic disease, you will speak the general verdict of the profession when you say that no special apprehension need be felt, yet do not be positive that you will be able to effect a radical and permanent cure, for the reasons already given. We do not entirely assent to the proposition that these neuroses are entirely harmless and do not in any way affect the vital expectancy of the individual, though, of course, possessing nothing like the gravity which attaches to organic disease of the heart. Experience of cases, which we cannot here detail, impress me with the conviction that a heart which is subject to these neurotic disturbances is not near so good or reliable as a vital machine as the healthy heart which does not possess this irritability.

*Cases Nos. II and III.*—These cases will be considered together, as they illustrate very perfectly the two types of *organic heart disease* which you will often meet with in your practice. No 2 we examined in your presence a week or two since, and though not present to-day, while the history is still fresh in your minds, we will again allude to it, and briefly sketch the clinical history and the physical symptoms elicited. On examination the patient, a colored woman 75 years of age, says that for a long time she has suffered from shortness of breathing, palpitation and frequently awakes at night oppressed and strangled for breath. You observe that she has a disposition to sigh at frequent intervals, is easily fatigued and has an aged and care-worn look, greater than even the number of her years should bring. These symptoms, or something nearly approaching them, might be producd by a fatty degeneration of the heart without any special obstruction to the blood current, so we proceed to investigate by the physical method. On inspection and palpation the apex beat of the heart was found to be some three-quarters of an inch to the left of the normal vertical line, drawn through the left nipple; percussion showed the area of cardiac dulness to be increased. On auscultation we detect a comparatively loud murmur which follows, or indeed, partially masks the first sound of the heart; the second sound is not well accentuated and is somewhat indistinct. On carefully moving the ear over the surface of the chest till we reach the level of the cartilage of the third rib at its point of junction with the sternum, we find the murmur clearer and most distinct here, but can be clearly distinguished throughout the area covered by the sternum. On applying the stethoscope over the carotid arteries, on either side of the neck, the murmur is still distinctly heard. We now have all the facts necessary to an accurate diagnosis. The murmur quickly following, treading on the heals of the first sound, as it were, is undoubtedly produced by a constriction of the aortic opening, which causes a partial obstruction to the onward flow of the blood into the aorta, and hence the murmur. The defective nature of the second sound is produced by a slight leakage from an imperfect closure of the diseased and thickened semi-lunar valves which guard the aortic opening. You may ask, if the murmur follows closely upon or partially replaces the first sound, may it not be due to at least a slight leak from imperfect closure of the aortic valves? This is not possible, as the area of greatest intensity is at the point of the chest walls where the normal second sound of the heart is loudest, viz: At level of the cartilage of the third rib, at the outer border of the sternum, for there the sound vibration of the murmur is conducted along the column of blood in the aorta, and can be distinctly noted in the cervical region, where the second sound of the heart and murmurs connected therewith are heard most distinctly.

*Case No. III.*—Mrs. A. J. McK. White, age 37. This patient which I now present before you is only of moderate intelligence, says that for the past two years she has suffered from shortness of breathing, cough and palpitation, that she frequently awakes at night with a choking sensation or difficulty of breathing. She says that she has suffered at times with pain in various parts of her body and occasionally has been laid up, but is not able to give any very clear account of her past history. On examination of the pulse we find it medium full and strong, but find it rather jerky and unsteady. On inspection and percussion we find the area of precordial dulness somewhat enlarged. On auscultation we hear a loud rasping murmur most intense near the apex of the heart and taking the place of the first sound of the heart. On making her separate the scapulæ by grasping the shoulders with each hand and exerting traction, drawing the shoulders forward and inward, and placing the ear below the angle of the left scapula, we detect the same murmur which we noted near the apex in front. As the first sound of the heart is produced by the flapping back of mitral valves and closure of the left auriculo-ventricular opening, we must believe that the loud rasping sound which we hear is due to a leakage through the imperfectly closed valves, the blood rushing through causing the valvular edges to vibrate and give forth the sound which you hear.

In the case of both No. 1 and No 2. the morbid sound is noted during the systole of the heart, or while the ventricles are contracting. For all practical purposes you may, at least until you become experts, confine your studies to the consideration of the left side of the heart. From what we have said as to the locality of the sounds in Nos. 1 and 2, you can have no dificulty in making a clear diagnosis as to the valves or opening of the heart which is involved. In addition to this we would call your attention to the relative age of the two subjects. No. 2 would even to the unscientific observer be classed as unmistakably in the "sere and yellow leaf," looking fully the age which she gives, or older, and it is among this class of persons that cases of this type are found. Degeneration and deterioration are written all over her, and the pathology of the disease is degeneration of atheromatous character, affecting the aorta and constricting its caliber, including the aortic opening at the cardiac extremity, it is essentially a disease of the aged. It is true that in the victims of syphilis, and occasionally in the comparatively young man you will meet with cases of this character, but even they look old and wasted beyond what their years should bring—prematurely aged. Murmurs of the character exhibited before you, usually assert themselves in the young and middle-aged, though it is true that no age is entirely exempt. Of all the exciting causes rheumatism is the most frequent, and in every case of rheumatic fever you must constantly be on your guard and daily examine the heart, as by the continual circulation of the poison in the blood which is believed to be of an acid nature, an inflammation of the lining of the heart, endocarditis, is developed and a stiffening and thickening of the mitral valves is the result. This we believe to have been the clinical history of the case before you. In recent cases it is often possible by the use of digitalis to give tone and volume to the hearts action, and by the use of alteratives, as potass. iodide, very minute doses of mercury, etc., long continued, to cause an absorption of the plastic material which has been thrown out and effect a cure either partial or entire. Another point you must bear in mind is that the loudest murmur does not always indicate the greatest danger to the patient, as a small quantity of blood forcibly urged through a small chink, between the edges of valves which are tense, may originate a loud note, while a large volume of blood may regurgitate through valves so incomplete that they offer scarcely any resistance and give forth very little sound. Of course the regurgitation of a portion of the blood calls for increased power on the part of the cardiac muscle, and the heart, if the general health be fairly good and the strain not too great, responds to the increased labor and becomes thicker and stronger, hypertrophied, but if the strain be too great the muscle stretches and gives way before it and dilatation of the heart ensues; the blood pressure is diminished and œdema, dropsy and all the ills of advanced heart disease assert themselves. But even in these cases you need not altogether despair; if the watery effusion be considerable administer hydragogue cathartics and drain it away. Put your patient on a systematic course of iron and digitalis, and in not a few cases you will be rewarded by seeing your patient grow stronger; his cardiac muscles, under the use of digitalis, act with more deliberation and effectiveness and the heart, which was yielding to dilatation, will become hypertrophied, the balance of the circulation will be restored and your patient, with care, may live many years in comparative comfort. There is nothing better attested, by the experiments of the physiologist and the experience of the profession, than the fact that digitalis does reduce the number of pulse beats, and thus give longer intervals of rest to the strained and overworked organ, but that each contraction is more efficient, and the volume of blood sent through the arteries is larger and stronger.

For No. II—Aortic constriction—there is only a gloomy future. First, there is not in these cases the reserve vitality which the young possess, which we can, by intelligent encouragement and assistance, enable to assert itself. In the second place, as we have seen, it is essentially a disease of the general system and associated with degeneration of structure throughout the body, fatty degeneration of cardiac muscles, etc. The best we can do for such cases is to guard them against over exertion, regulate the diet, giving a liberal supply of simple, easily digested food, and giving small doses of digitalis combined with nux vomica. These are the cases which are liable to end in sudden death, and fatal syncope may follow sudden excitement or strong muscular effort.

